# Psychometric Properties of the 34-Item Short-Form Supportive Care Need Survey (SCNS-SF34) Scale in the Malaysian Cancer Healthcare Context

**DOI:** 10.3390/ijerph18179403

**Published:** 2021-09-06

**Authors:** Nizuwan Azman, Lei Mee Thien, Mohammad Farris Iman Leong Abdullah, Noorsuzana Mohd Shariff

**Affiliations:** 1 Unit of Biostatistics and Bioinformatics, Division of Research and Networking, Advanced Medical and Dental Institute, Universiti Sains Malaysia, Bertam 13200, Penang, Malaysia; nizuwan@usm.my; 2 School of Educational Studies, Universiti Sains Malaysia, Minden 11700, Pulau Pinang, Malaysia; thienleimee@usm.my; 3 Lifestyle Science Cluster, Advanced Medical and Dental Institute, Universiti Sains Malaysia, Bertam 13200, Penang, Malaysia; farris@usm.my

**Keywords:** assessment, cancer, psycho-oncology, psychometric properties, SCNS-SF34, supportive care needs

## Abstract

(1) Background: This study aimed to assess the psychometric properties of the Malay version of SCNS-SF34 among Malaysian cancer patients. (2) Methods: This cross-sectional study involved 171 cancer patients. Data were collected using the structured five-factor survey via telephone call or face-to-face interviews. The internal reliability and the construct validity of SCNS-34M were analysed using principal component analysis with varimax rotation. (3) Results: The health system and information need (HSI) was the domain with the highest mean score (2.73 ± 0.88), followed by patient care and support needs (2.16 ± 0.90), as well as physical and daily living needs (1.99 ± 0.98). The confirmatory factor analysis indicated a moderate model fit for RMSEA with 0.070, TLI = 0.911 and CFI = 0.924. (4) Conclusions: The SCNS-SF34M was found to be a conceptually applicable and culturally appropriate scale in measuring the supportive care for cancer patients within the Malaysian context.

## 1. Introduction

According to the World Health Organization (WHO), cancer ranks as the first or second leading cause of death before the age of 70 years in 91 out of 172 countries globally [1]. In the recent cancer statistics released in 2020, an estimated 19.3 million new cases of cancer and 10 million cases of cancer deaths were reported worldwide. Furthermore, the number is expected to rise to 28.4 million cases in the next two decades. Half of all the cancer cases and more than half (58.3%) of cancer deaths are estimated to have occurred in Asia in 2020, where 59.5% of the global population resides. Of all the cancer types, breast cancer is the most commonly diagnosed cancer, with an estimated incidence of 2.3 million new cases (11.7%), followed by lung cancer (11.4%) [2]. Due to the physical and psychological difficulties throughout cancer treatments, there is increasing attention among researchers and physicians on the unmet needs of supportive care for cancer patients and survivors. The provision of supportive care has been significantly associated with patients’ survival, rehabilitation, quality of life and the prioritisation of medical service utilisation [3,4,5].

The Supportive Care Needs Survey (SCNS) is an instrument used to assess the perceived needs of adults diagnosed with cancer. The initial version of the survey in the English language contained 59 items that covered five specific needs pertaining to psychological, health system and information, physical and daily living, patient care and support, as well as sexuality needs [6,7]. The practicality of the survey was then further improved and re-introduced as the 34-item short-form Supportive Care Need Survey (SCNS-SF34). The new version has been proven to have better internal validity and reliability than the original version [8]. Since then, the short, improved version has been widely used in many countries and translated into different languages, such as in Chinese [9,10,11], Korean [12], Japanese [13], French [14], German [15], Mexican [16] and Turkish [17]. The reliability of the translated version of SCNS-SF34 varied across cultures with the Cronbach alpha values ranging from 0.64 (Chinese version) to 0.94 (Germany version). These values are comparable to those of the original English version (from 0.86 to 0.96) [8].

Previous translation of the survey showed variability in terms of the factorial structure used in different settings. The study conducted in Turkey has demonstrated that the four-factor was the best model to adapt in Turkish society for the determination of the supportive care needs of breast cancer patients [17]. It was supported by a study by Au et al., where the four-factor was the best final model. They revealed that, in Asian communities, communicating care and concern can sometimes take priority over the search for information [9]. Meanwhile, the studies in Korea, Japan, France, Germany and Mexico were still maintaining the five-factor as their best final model in assessing the psychometric properties among cancer patients in their countries [12,13,14,15,16]. Hence, more studies are needed to show the factorial structure of the survey across different cultures, as there is still no consensus made referring to the best factorial structure for this respective survey.

Due to the increasing cancer incidence and mortality in the region [2], there have been growing interests in assessing the unmet supportive care needs of cancer patients in Malaysia and other Malay-speaking populations in the neighbouring countries [18,19]. Despite so, there is a lack of psychometric assessment of the Malay-translated version of SCNS-SF34. Most of the previous studies relied heavily on the English version. As a result, the responses from the study participants may vary based on their understandings of the survey. Thus, these studies failed to take into account the effects of the cultural and contextual differences in the Malay-speaking populations. In view of this, we aimed to determine the psychometric properties of the Malay version of the 34-item short-form Supportive Care Needs Survey (SCNS-SF34M) among cancer patients in Malaysia and to recommend a culturally practical and relevant tool for assessing the quality of supportive care from the perspective of Malaysian cancer patient population.

## 2. Materials and Methods

### 2.1. Sampling and Data Collection Procedure

This cross-sectional study was conducted in a tertiary governmental cancer institute in Penang, Malaysia, from 1 June 2018 to 30 June 2019. It was part of a multi-centre prospective cohort study that assessed the unmet supportive care needs among cancer patients throughout their treatment trajectory in Penang, Malaysia (PenBCNeeds Study) [20]. Patients diagnosed with any type of cancer for less than a year, aged 18 years old and above, with the basic level of Malay language proficiency, willing and able to complete the interview and who attended the outpatient oncology clinic were eligible to participate in the study. On the contrary, patients who were critically ill, mentally unstable, or did not consent were excluded from the study. By using the general rule of five-respondents-to-one-item ratio [20], a total of 171 patients were interviewed either via face-to-face or telephone interview. Convenience sampling was used in selecting the study participants. The eligible patients were identified through the clinic appointment database two days prior to their appointment day. On the day of the appointment, patients were approached by the researchers during the waiting time. Each participant was given explicit information about the purpose of the study. Informed consent was obtained in the form of verbal approval. Face-to-face interviews were carried out in the clinic on the same day for those who agreed to stay for the interview. However, those who were unable to stay for the interview were given the flexibility to choose another time and date of their convenience to be interviewed over the phone. From 171 patients involved, a total of 17 patients was identified and agreed to be interviewed through the phone call by the researchers and research assistant. The research assistant had been trained by the research team prior to data collection to ensure standardisation of technique of conducting interviews.

### 2.2. Ethical Consideration

Ethical approval was obtained from the Medical Research and Ethics Committee, Ministry of Health Malaysia (NMRR-19-268-45809 IIR) and the Human Research Ethics Committee of Universiti Sains Malaysia (USM/JEPeM/17100443). Each of the participants was given explicit information about the study and their informed consent was obtained by researchers before the interview. The participation was on a voluntary basis. Each participant was given the full autonomy to withdraw from the study at any time. All the information and data obtained were treated with full confidentiality.

### 2.3. Instrumentation

This study adopted the original version of the 34-item short-form Supportive Care Need Survey (SCNS-SF34). It consists of five domains assessing (i) physical and daily living needs (5 items), (ii) psychological needs (10 items), (iii) sexuality needs (3 items), (iv) patient care and support needs (5 items) and (v) health systems and information needs (11 items). Participants scored their needs for each item using a five-point Likert scale ranging from 1 (no need, not applicable) to 5 (some need, high need for help) [7].

In the first phase, the translation of the items in the questionnaire into the Malay language was performed by four independent language experts with good proficiency in both English and Malay languages, with two of them from the research team. Next, the forward–backward translation (Malay to English and back to Malay) was performed by another independent language expert who was not associated with this study. To ensure the conceptual equivalence of the translated items, both sets of English and Malay versions were subsequently compared with the original version by another four experts in cancer research who were fluent in both languages, with two experts from the research team. Subsequently, for face validity, pre-testing was carried out on ten cancer patients to identify any ambiguous words or statements [20]. No firm rules exist for the recommended sample size for item-rating tasks, but researchers typically use sample sizes ranging from 10 to 30 [21,22,23,24]. This had also been practiced in the study performed by Ibrahim et al., in 2019, where a similar number of patients was involved during pre-testing [25]. During the pre-test, two items were modified to ensure the meaning and content of the questionnaire was well understood by the respondents. The example of the item which was improved was item number 17, “Concerns about the worries of those close to you”, and item 28, “Being informed about your cancer which is under control or diminishing (that is, remission)”. These two statements were modified to ensure they were clear and easy to understand. In our current study, no content validity index was performed but we used the qualitative analytic methods to review the content validity of the translated version of the questionnaire from the review of our expert panel. The feedback given by the expert to every item in the survey was used to improve the questionnaire and assessment of the content validity [20]. Following that, the necessary amendment was made to the translated version before the final version was used for data collection.

### 2.4. Data Analysis Procedure

The participants’ characteristics and other descriptive findings were presented as frequency and percentage for categorical variables or mean and standard deviation for continuous variables. The reliability of items was determined using the Cronbach’s alpha (CA) coefficient. The item-to-total correlation was used to determine the internal consistency among items, whereby values of more than 0.70 were considered as acceptable [26]. Additionally, the exploratory factor analysis was used to assess the factorial validity of the five-factor model with the aim of reducing the number of items into a smaller dimension, so that the data could be simplified into a better model. Maximum likelihood and principal component analysis with varimax rotation were used to determine the best factor to be included in the model. Furthermore, discriminative validity was also used to discriminate between age and sex groups. This was based on translation studies conducted previously, that showed the discriminative effect of the two variables [11,12]. For this purpose, the independent *t*-test and one-way ANOVA were used to compare the means of different factors/groups. The chi-square test was supplemented with the root mean square error of approximation (RMSEA), comparative fit (CFI) and the Tucker–Lewis (TLI) indices. RMSEA values less than 0.06–0.08 with a 95% confidence interval were deemed as an acceptable fit [27]. The general cut-off criterion for CFI and TLI was 0.90 for acceptance, respectively [28,29]. The AMOS (Analysis of Moment Structures) IBM SPSS version 23 (IBM Corp., Armonk, NY, USA) was used for confirmatory factor analysis, while SPSS Statistics version 27 (SPSS Inc., Chicago, IL, USA) was used for exploratory factor analysis and other descriptive analysis. The *p*-value was set at 0.05. All of these activities were carried out in the second phase of the study.

## 3. Results

### 3.1. Participants’ Characteristics

A total of 171 participants was involved in the study with a 100% response rate. The majority of the patients were female (90.1%). The mean age of the respondents was 52.21 years with a range between 27 and 76 years. At least 30% of the patients had received either chemotherapy, radiotherapy, surgery, or hormonal therapy, or a combination of any of these cancer treatments, prior to the study. Breast cancer was the most common cancer, involving 74.3% of the patients. However, out of the 171 patients, 14.6% did not provide complete information regarding their diagnosis (Table 1).

### 3.2. Reliability

The internal consistency for the five-factor model was good, as the Cronbach’s alpha value exceeded the acceptable value of 0.7 for all five domains. The PCS domain was observed to have the highest Cronbach’s alpha value of 0.929, followed by the PDL and HIS with both domains having recorded the same Cronbach’s alpha value of 0.918. The S and P domains reported Cronbach’s alpha values of 0.901 and 0.883, respectively. The reliability of the five-factor SCNS-SF34M model is presented in Table 2.

### 3.3. Factor Analysis

#### 3.3.1. Exploratory Factor Analysis

The Kaiser Meyer-Olkin (KMO) measure of sampling adequacy test and Bartlett’s test of sphericity were conducted prior to the exploratory factor analysis (EFA) to evaluate the factorability of the items. The KMO value was 0.878 and the significant value for Bartlett’s test of sphericity was less than 0.05, thus indicating a good outcome of EFA for the obtained dataset. A total of five factors were extracted and rotated. The factor loading of all the 34 items and total variance explained for each dimension are shown in Table 2. All components with eigenvalues of more than one were retained using parallel analysis and scree plot. All the items recorded an acceptable factor loading of >0.3. Items with a loading factor of more than ±0.3 would be considered as having an acceptable loading factor [30]. Item number 17 had a factor loading of less than 0.4 for all factors. The study by Yusoff et al., in 2019, suggested a factor loading with a low cut-off value of 0.3 during the EFA stage can be considered [31]. Any item with low factor loading was kept with its original factor to maintain the originality of the content.

In addition, the domain of health system and information needs (HSI) was observed to have the highest mean score (2.73 ± 0.88), followed by the patient care and support needs (PCS) domain (2.16 ± 0.90). The lowest mean was recorded for the sexuality needs (S) domain with a mean value of 1.90 ± 0.80. The floor score and ceiling score in this study ranged from 15.2% to 44.0% and from 0.6% to 10.4%, respectively (Table 2).

#### 3.3.2. Confirmatory Factor Analysis

**Figure 1:**
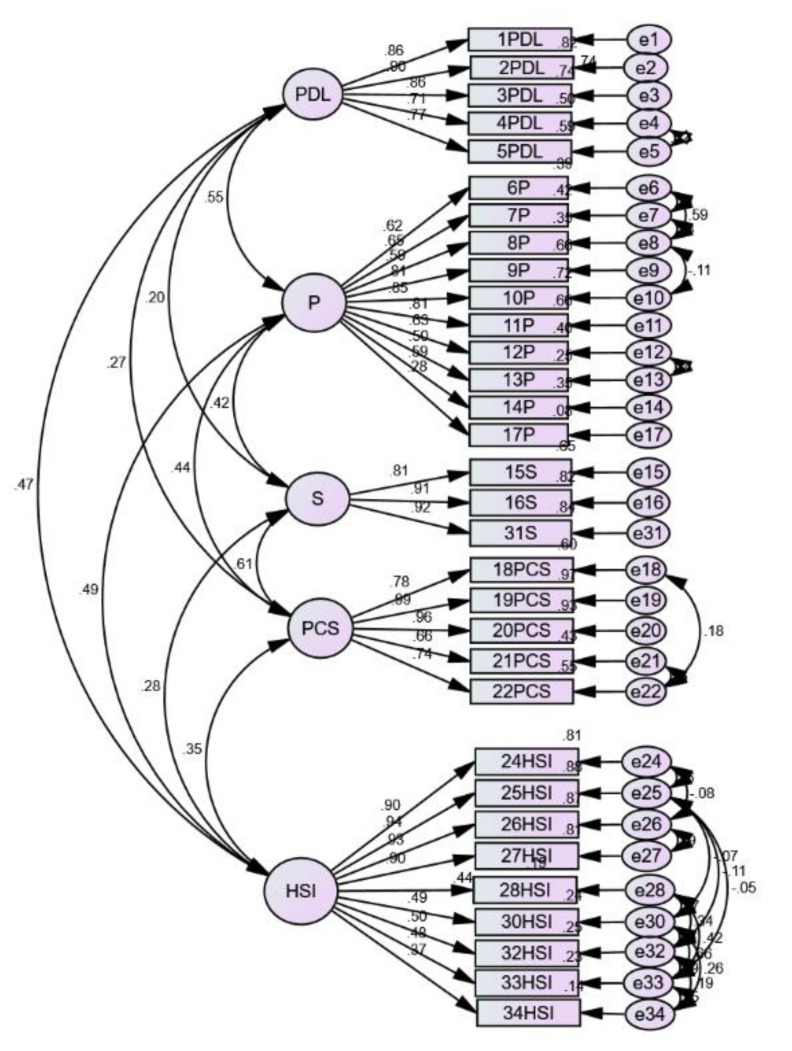
Results of structural equation model analysis for the short-form Supportive Care Need Survey Malay version (SCNS-SF34M). Physical and daily living needs (PDL), psychological needs (P), sexuality needs (S), patient care and support needs (PCS) and health systems and information needs (HSI).

Figure 1 shows the standardised factor loadings (standardised regression weights) for each item in the five-factor model for CFA of SCNS-SF34M. One item (number 17P) had standardised factor loading values of less than 0.3. However, the items were retained in the measurement model because they were considered to be acceptable in terms of content and were reviewed and deemed important by the expert during the content validity process [20]. Next, modification indices (MIs) were referred to while examining the presence of redundant items in the measurement model.

An MI of less than 15 was considered acceptable for all items [32,33]. Two options can be considered in addressing this problem. The first option is to delete one of these two redundant items and respecify the measurement model. Another option is to set these two correlated errors to be “free parameter estimate” and respecify the measurement model [33].

In this study, the second option was chosen. Several MIs that exceeded the specified limit were identified. The first part involved the correlation analyses of e33–e28 (47.016), e33–e34 (39.219), e32–e28 (30.206), e32–e34 (18.462), e32–e33 (69.393), e30–e28 (52.045), e30–e34 (30.413), e30–e33 (83.615), e30–e32 (43.664), e26–e27 (59.785), e25–e33 (15.919), e25–e32 (19.123), e25–e30 (16.650), e24–e26 (15.056), e24–e25 (17.015), e21–e22 (57.360), e18–e22 (15.921), e12–e13 (23.165), e8–e10 (16.040), e7–e8 (36.026), e6–e8 (38.156), e6–e7 (71.234) and e4–e5 (40.905). Then, the analysis was repeated. The final result indicates that none of the errors was correlated, except for e17–e16 (15.028) and e13–e25 (17.398); however, the errors could be attributed to the different domains.

Hair et al. suggested that model fit can be decided by at least a minimum of three different indices [34]. In this study, the final five-factor model of the SCNS-34M showed an acceptable fit and the fitness indices were as follows: χ^2^ (423) = 780.14 (*p* < 0.001), RMSEA = 0.070, CFI = 0.924 and TLI = 0.911.

### 3.4. Discriminant Validity

Table 3 reveals the low to moderate significant positive correlations between all the five domains, ranging from *r* = 0.20 between the physical and daily living (PDL) and sexuality (S) domains to *r* = 0.57 between the patient care and support needs (PCS) and health systems and information needs (HSI) domains. The highest correlation value was recorded between PCS and HIS, with moderate strength (*r* = 0.569), followed by the PCS and S domains (*r* = 0.552). Meanwhile, the correlation between other domains was positive but at low to moderate strength (from *r* =0.200 to *r =* 0.506).

The SCNS-SF34M scores for each domain were then compared by known group differences (Table 4). The PDL and HSI domains indicated a significant mean difference by gender with *p*-values of 0.029 and 0.010, respectively. Male participants scored higher in the PDL domain than women. In contrast, the female group scored higher than the male group in the HSI domain. The other domains did not show any significant difference by gender. The Fornel–Larcker criterion also indicated an acceptable discriminant validity assumption with the square root of AVE (PDL = 0.823, *p* = 0.836, S = 0.700, PCS = 0.879 and HIS = 0.654) that was more than the correlation of the constructs stated in Table 3 [35].

Meanwhile, there was a significant mean difference by age group for their sexuality needs, e.g., the S domain (*p*-value = 0.004) (Table 5). Further analysis using the Bonferroni post-hoc test showed that patients between 40 and 49 years old and more than 50 years old varied significantly in the S domain (*p*-value = 0.009).

## 4. Discussion

Since the 34-items short-form Supportive Care Needs Survey (SCNS-SF34) was developed, it has been translated and validated in many other languages to further confirm the psychometric properties of the translated version among a mixed group of cancer patients in different cultural settings [9,12,13,14,15,17]. To the best of our knowledge, this was the first study to assess the factorial structure of the Malay version of the SCNS-SF34 involving Malay-speaking cancer patients with various diagnoses using the confirmatory factor analysis. A similar assessment has only been conducted in other languages [8,15].

The study findings revealed that the most prevalent unmet needs for cancer patients were information regarding the status of their cancer (i.e., either under control or diminished), pleasantness of treatment condition in a hospital or clinic and things they can do to help themselves get better. All these three items were categorised under the same domain, i.e., health system and information (HIS) needs. This finding is consistent with a study in China, whereby the same domain recorded the highest prevalence in terms of unmet supportive care among cancer patients [11,36]. Another study by Hwang and Park (2006) in Korea has also proven that in most of the Asian developing countries, HIS was the main domain associated with the highest degree of unmet needs [12]. On the other hand, Edib et al. reported contrasting findings in which the main concern was Psychological needs, such as uncertainty about the future, fears about cancer spreading, feelings of sadness, feeling about death and dying, concerns about those close to the patient, worry that the result of treatment is beyond control and feeling down or depressed [18].

Furthermore, this study shows that the SCNS-SF34M has an acceptable fit and good reliability. The initial five domains of factors were examined using the principal confirmatory analysis (PCA). In 2017, Ozbayir et al. published the Turkish version of the SCNS-SF34 in which several items were removed and the survey was reduced from five into four main domains, namely health care services and informing, psychology, sexuality and daily life [17]. The KMO in their study (0.840) was lower than the current study (0.878). Meanwhile, the SCNS-SF34-Fr was also associated with a fit and fairly acceptable outcome with RMSEA (0.076), CFI (0.96) and TLI-NNFI (0.96) all within an acceptable range [14]. In comparison, the CFI and TLI domains in our study showed lower values than the acceptable range and only the RMSEA value was within the acceptable range (0.070). Our study results are aligned with Li et al., whereby their models also did not fulfil the minimum criterion, except for the RMSEA [10].

Even though our findings provided support to retain the original factor structure, Au et al. made different observation whereby their analysis of SCNS-SF34-C resulted in the removal of one domain, with only health system, information and patient support, psychological need and physical and daily living, as well as sexuality, remained in the translated version [9]. The Cronbach’s alpha for the physical and daily living domain in the SNCS-SF34-C had the lowest value of 0.75. However, it was retained, as the value was still above the acceptance margin. All the other domains indicated good internal consistency with values from 0.82 to 0.92 [9]. On the contrary, the internal consistency for the domain of physical and daily living of the SCNS-SF34M in our study exhibited a much higher Cronbach’s alpha value of 0.918. A similar outcome was observed by Ozbayir et al., whereby their SCNS-SF29^Tr^ also reported four significant domains. The four domains were established via language validity, content validity and structure validity that fit the Turkish culture [17]. In their study, the Cronbach’s alpha obtained for the four domains indicated high internal consistency with values ranging from 0.83 (daily life), to 0.88 (psychology), sexuality (0.91) and health care service and informing (0.95), as similarly recorded for the SCNS-SF34M in this study, i.e., from 0.883 to 0.929 [17]. The variability in reliability of SCNS-34 used in different settings can be related to the inter-individual variability in a sample and testing in different (e.g., patient) samples [37].

On a similar note, the German-translated version, SCNS-SF34-G, was found to show different mean values in specific domains for patients with different sex and age groups [15]. In our study, the SCNS-SF34M was able to discriminate between gender but only on the domain health system and information needs and physical and daily living needs. From the result, female patients demonstrate a higher number of needs for the domain health system and information needs than male patients and this could be due to a number of patients involved where female patients were the majority in this study. This finding is consistent with the results from the study in Korea and Hong Kong, where the domain health system and information ranked highest among other domains [9,12]. The systematic review by Fiszer et al. has also suggested that Asian women were reported higher in term of informational needs than Western women [38]. The culture of how health care providers treat the patients also may differ among each country, especially among Western and Asian countries. These findings contradict with a study in German, where the physical daily living needs was significantly correlated with patients with advanced stages [15]. It was supported by a study in Korea, where patients receiving or having received chemotherapy were more likely to report some needs in the physical and daily living needs domain [5].

Besides that, in our study, the sexuality needs domain, again, significantly varied by patient’s age group; this is parallel to a study in Malaysia in 2016, where the domain reported significant differences across the age groups (*p*-value = 0.001). This might be due to the attitude across ages, with younger patients more likely to vocal out their concerns on sexual needs [19]. This in line with a study in Australia, where the patients with age between 31 and 60 years had a better approach on sharing their experience on sexual life than other age groups [39]. Specifically, the analysis of SCNS-SF34M revealed that patients below 40 years old reported a higher mean value than other age groups and the finding coincided with SCNS-SF34-G [15].

Furthermore, a study by Bredart et al. revealed a high correlation between the domains of psychological needs and PDL [14]. A similar finding was reported in another two studies conducted in Hong Kong and Japan [9,13]. However, the results from the above-mentioned studies contradicted our result, whereby a weak correlation was observed between the PDL domain and the other domains, namely, sexuality needs and patient care and support needs, as well as health system and information. In Mexico, the sexual need domain was less relevant to their population and this approach might be due to no studies having been conducted [16]. On a slightly different note, our study observed a moderate correlation between PDL and psychological needs. This is in line with Lehman et al., in which a good and moderate correlation was noted between the domains of PDL and psychological needs in SCNS-SF34-G [15].

In terms of gender, Choi et al. showed that female patients suffered significantly more in terms of coping with their daily lives, experiencing psychological distress and navigating the health system [11]. The research by Lehmann et al. also found that women recorded a higher mean score in terms of psychological and physical needs, compared to men who scored higher for sexuality needs [15]. However, these findings contradicted our study in which men were found to be more likely to have higher psychological needs from the aspects of feeling sad, anxious, depressed and fearful about cancer spreading. However, this difference was not statistically significant. A similar outcome was observed for the PDL domain, whereby male patients had a significantly higher mean score than females. In addition, Davis et al. emphasised the need to improve gender-specific patient-centred care in cancer healthcare [40]. Nevertheless, in this study, there was no significant mean difference between genders for the domains of psychological needs and sexuality needs, as well as patient care and support needs. In Korea, the younger age had a greater psychosocial impact than elder age. It can be explained due to different attitudes towards reporting unmet needs between younger and older patients even though some studies indicate the greater stress in younger age [12]. Although, in our Malaysia context, sexuality needs were not openly discussed because of cultural value, the health care provider should include them as part of their routine care. This in line with a study in Korea where the sexual needs were less cared about. The spouse of the patients should play their role in improving their sexual life [5].

Next, the internal consistency for the SCNS-SF34M in this study indicated that all domains had good and high reliability as the Cronbach’s alpha coefficient was more than 0.800. In Germany, Lehmann et al. proved that the SCNS-SF34-G version provided excellent outcomes tailored to the patient’s needs. Moreover, all the Cronbach’s alpha values in their study, e.g., health system and information (0.95), psychological (0.94), physical and daily living (0.85), patient care and support (0.89) and sexuality (0.82) showed good internal consistency [15]. In line with our study, the initial version of the SCNS-SF34 by Boyes et al. also proved that all five domains performed well in terms of internal consistency, with a Cronbach’s alpha of more than 0.860 [8]. Meanwhile, the Chinese version of the SCNS-SF34 demonstrated good internal consistency, with a Cronbach’s alpha of more than 0.700 for all domains (health system and information (0.855), psychological (0.87), physical and daily living (0.74) and patient care and support (0.76), except for sexuality (0.64) [11]. Even though our study performed well in all five domains, the psychological domain showed the lowest mean score for internal consistency. Thus, further research might be warranted to improve the survey instrument.

Nevertheless, the SCNS-SF34M was shown to have a good outcome with exploratory factor analysis, as the Kaiser-Meyer-Olkin (KMO) was 0.878 and all the factor loadings were above the acceptable value (more than 0.3) [30]. Our finding is in line with the other five-factor SCNS-SF34 survey conducted in Germany by Lehmann et al. [15]. Besides, the SCNS-SF34M version is also compatible with the four-factor models in the SCNS-SF34 Turkish version (SCNS-SF29^Tr^) and the SCNS-SF34 Chinese version (SCNS-SF34-C) [9,17].

Despite the above findings, this study has a few limitations. Firstly, the sample size in our study was relatively small, compared to other validation studies of the SCNS-SF34. Furthermore, the participants in this study were recruited from a single institution. Thus, this study was also limited in terms of the variability of patients’ experience from the aspects of their cancer treatment and care environment. Therefore, the study findings might not be generalisable to the Malaysian cancer population. Besides that, a broader range of factors in terms of education level, sociodemographic background, cancer duration and cancer stage should be studied to obtain better validation outcomes. In addition, cultural factors and language proficiency were also the main barriers in implementing this study. Most of the patients were reluctant in providing the best answers, especially when it comes to the sexuality aspect, although good internal consistency of this domain was still reported.

In short, with good internal consistency obtained for all five domains, the SCNS-SF34M is recommended as a practical and reliable tool to gather data pertaining to the supportive care needs among the Malaysian cancer patients’ population. Therefore, it is hoped that surveys can be performed with the SCNS-SF34M to provide necessary information on the cancer patients’ current and future needs to guide the routine clinical practices for the healthcare professionals from both private and public sectors. Lastly, following the validation and proven reliability of the SCNS-SF34M, it can be applied as a practical and reliable tool in future researchers that aim at identifying the needs and requirements for developing a supportive care ecosystem needed by the Malay-speaking cancer patients’ population.

## 5. Conclusions and Future Recommendation

In light of the study findings, SCNS-SF34M confers acceptable psychometric properties and good reliability in assessing the specific needs of cancer patients. It is also compatible with the original English version of the SCNS-34. Future studies should include other cancer patients in Malaysia to further confirm the psychometric properties of the SCNS-SF34M so that its use can be expanded for future clinical or research usage, especially in the cancer healthcare context.

## Figures and Tables

**Table 1:** Characteristics of the participants (*n* = 171).

Patients’ Characteristics	*n* (%)
Gender	
Male	17 (9.9)
Female	154 (90.1)
Age (mean ± SD)	52.21 ± 9.05
Max (years)	76
Min (years)	27
Diagnosis *	
Breast cancer	127 (74.3)
Colorectal cancer	6 (3.5)
Cervical cancer	3 (1.8)
Lung cancer	2 (1.2)
Nasopharyngeal cancer	2 (1.2)
Lymphoma cancer	1 (0.6)
Oral cancer	1 (0.6)
Skin cancer	1 (0.6)
Synovial sarcoma	1 (0.6)
Throat cancer	1 (0.6)
Treatment received	
Chemotherapy	51 (29.8)
Radiotherapy	49 (28.7)
Surgery	50 (29.2)
Hormone	2 (1.2)

* 25 patients (14.6%) did not state the type of cancer diagnosis.

**Table 2:** SCNS-SF34 Malay items (mean and standard deviation) and factor loadings in exploratory factor analysis (*n* = 171).

Factor and Item Number	Mean ± Standard Deviation	Floor Score (%)	Ceiling Score (%)	Factor Loadings	Cronbach Alpha	Eigenvalues	Total Variance Explained (%)
Physical and daily living needs (PDL)	1.99 (0.98)	44.0%	4.3%				
Item 1	1.94			0.801	0.918	35.698	34.864
Item 2	2.05			0.838			
Item 3	1.71			0.831			
Item 4	2.11			0.820			
Item 5	2.16			0.831			
Psychological needs (P)	1.92 (0.54)	30.6%	0.7%				
Item 6	1.64			0.628	0.883	12.449	11.669
Item 7	1.45			0.649			
Item 8	1.74			0.640			
Item 9	2.20			0.686			
Item 10	2.01			0.729			
Item 11	2.09			0.704			
Item 12	2.11			0.627			
Item 13	2.24			0.574			
Item 14	1.89			0.662			
Item 17	1.80			0.334			
Sexuality needs (S)	1.90 (0.80)	38.8%	0.6%				
Item 15	1.96			0.578	0.901	7.981	7.240
Item 16	1.74			0.527			
Item 31	1.99			0.623			
Patient care and support needs (PCS)	2.16 (0.90)	29.8%	1.8%				
Item 18	1.84			0.744	0.929	6.168	5.287
Item 19	2.18			0.806			
Item 20	2.22			0.824			
Item 21	2.12			0.778			
Item 22	2.46			0.844			
Health system and information needs (HSI)	2.73 (0.88)	15.2%	10.4%				
Item 23	2.81			0.459	0.918	4.823	4.048
Item 24	2.42			0.812			
Item 25	2.44			0.834			
Item 26	2.57			0.796			
Item 27	2.49			0.805			
Item 28	3.18			0.755			
Item 29	3.23			0.800			
Item 30	2.92			0.803			
Item 32	2.58			0.598			
Item 33	3.15			0.725			
Item 34	2.25			0.520			

Extraction method: principal component analysis; Cronbach’s alpha = 0.942; Kaiser-Meyer-Olkin = 0.878; significance of Bartlett’s test of sphericity < 0.05.

**Table 3:** Correlations among the five factors.

Domain	PDL	P	S	PCS	HSI
PDL					
P	0.506 **				
S	0.200 **	0.392 **			
PCS	0.282 **	0.416 **	0.552 **		
HSI	0.387 **	0.454 **	0.455 **	0.569 **	

** Correlation is significant at the 0.01 level (2-tailed); PDL, Physical and daily living needs; P, psychological needs; S, sexuality needs; PCS, patient care and support needs; HSI, health system and information needs.

**Table 4:** Comparison of gender with SCNS-SF34 Malay domains items.

Domain	Mean (SD)		*p*-Value *
	Male	Female	
Physical and daily living needs (PDL)	2.48 (0.98)	1.94 (0.97)	0.029 **
Psychological needs (P)	2.01 (0.59)	1.91 (0.53)	0.443
Sexuality needs (S)	1.65 (0.58)	1.93 (0.82)	0.172
Patient care and support needs (PCS)	1.99 (0.81)	2.18 (0.91)	0.400
Health system and information needs (HSI)	2.33 (0.60)	2.78 (0.90)	0.010 **

SD, Standard deviation; * Independent *t*-test was performed; ** Significant level < 0.05.

**Table 5:** Comparison of age group with SCNS-SF34 Malay domains items.

Domain	Mean (SD)			*p*-Value *
	<40 Years	40–49 Years	≥50 Years	
Physical and daily living needs (PDL)	2.11 (1.03)	1.87 (0.87)	2.10 (1.02)	0.491
Psychological needs (P)	1.89 (0.30)	2.01 (0.63)	1.88 (0.56)	0.552
Sexuality needs (S)	2.52 (0.90)	2.40 (0.69)	1.94 (0.71)	0.004 **
Patient care and support needs (PCS)	2.86 (0.76)	2.40 (0.95)	2.23 (0.92)	0.206
Health system and information needs (HSI)	3.05 (0.71)	3.06 (0.96)	2.98 (0.86)	0.899

SD, Standard deviation; * One-way Analysis of Variance (ANOVA) was performed; ** Significant level < 0.05.

## Data Availability

The data that support the findings of this study are openly available in (Mendeley Data) at http://dx.doi.org/10.17632/cpyzxvv2gs.1, accessed on 19 April 2021. The questionnaire presented in this study are available on request from the corresponding author. The data are not publicly available due to their containing information that could compromise the privacy of research participants.
